# Ammonia and ethanol detection via an electronic nose utilizing a bionic chamber and a sparrow search algorithm–optimized backpropagation neural network

**DOI:** 10.1371/journal.pone.0309228

**Published:** 2024-12-03

**Authors:** Yeping Shi, Yunbo Shi, Haodong Niu, Jinzhou Liu, Pengjiao Sun

**Affiliations:** 1 The Higher Educational Key Laboratory for Measuring & Control Technology and Instrumentation of Heilongjiang Province, Harbin University of Science and Technology, Harbin, China; 2 Electronics and Communication Engineering School, Jilin Technology College of Electronic Information, Jilin, China; 3 Heilongjiang Province Key Laboratory of Laser Spectroscopy Technology and Application, Harbin University of Science and Technology, Harbin, China; 4 National Experimental Teaching Demonstration Center for Measurement and Control Technology and Instrumentation, Harbin University of Science and Technology, Harbin, China; Federal University of ABC, BRAZIL

## Abstract

Ammonia is widely acknowledged to be a stressor and one of the most detrimental gases in animal enclosures. In livestock- and poultry-breeding facilities, a precise, rapid, and affordable method for detecting ammonia concentrations is essential. We design and develop an electronic nose system containing a bionic chamber that imitates the nasal-cavity structure of humans and canines. The sensors are positioned based on fluid simulation results. Response data for ammonia and ethanol gases and the response/ recovery times of an ammonia sensor under three concentrations are collected using the electronic nose system. Response data are classified and regressed using a sparrow search algorithm (SSA)-optimized backpropagation neural network (BPNN). The results show that the sensor has a relative mean deviation of 1.45%. The ammonia sensor’s output voltage is 1.3–2.05 V when the ammonia concentration ranges from 15 to 300 ppm. The ethanol gas sensor’s output voltage is 1.89–3.15 V when the ethanol gas concentration ranges from 8 to 200 ppm. The average response time of the ammonia sensor in the chamber is 13 s slower than that of the sensor directly exposed to the gas being measured, while the average recovery time is 19 s faster. In tests comparing the performance of the SSA-BPNN, support vector machine (SVM), and random forest (RF) models, the SSA-BPNN achieves a 99.1% classification accuracy, better than the SVM and RF models. It also outperforms the other models at regression prediction, with smaller absolute, mean absolute, and root mean square errors. Its coefficient of determination (R^2^) is greater than 0.99, surpassing those of the SVM and RF models. The theoretical and experimental results both indicate that the proposed electronic nose system containing a bionic chamber, when used with the SSA-BPNN, offers a promising approach for detecting ammonia in livestock- and poultry-breeding facilities.

## 1. Introduction

Ammonia is a hazardous gas that poses significant risks to human health and environmental quality. In livestock- and poultry-breeding areas, animals’ diet and excrement release ammonia into the environment [1–3]. The environmental protection agency (EPA) of the United States suggests that human exposure to 25 ppm of ammonia for one hour or 50 ppm for 10 min, could cause mild to moderate irritation of the upper airway, headaches, nausea, and decreased productivity. Animals exposed to an environment containing 100 ppm of ammonia can experience sneezing, salivation, and mucosal discomfort [4–6]. Therefore, it is crucial to effectively detect the volume concentration of ammonia gas in livestock- and poultry-breeding areas.

Gas-detection technologies in livestock and poultry facilities must feature a rapid response, good repeatability, and high sensitivity. Traditional electrochemical [7–9], spectroscopic [10–12], and analytical [13–15] ammonia detection methods are limited to laboratory settings owing to their complexity and high cost. These methods also tend to suffer from lower detection accuracy and generally do not offer real-time data. In contrast, electronic noses have advantages such as high sensitivity, fast response, and small size. Consequently, they have played a crucial role in gas detection for environmental monitoring [16–18]. Electronic noses mimic the operating principles of human olfactory cells, employing various sensors and biomimetic technologies to detect gases and odors rapidly, accurately, reliably, and repeatably. An electronic nose detects "gas fingerprint" information through a multi-sensor array and applies a pattern recognition technique. Electronic noses can detect various gas samples and identify the gas species to be measured and statistically project the desired gas concentration using an intelligent algorithm, unlike the electrochemical technique [7–9]. Electronic nose technology can eliminate diverse human errors and the objective monitoring results are unlike spectral detection [10–12]. When compared with instrumental analytical techniques, electronic nose technology is advantaged by miniaturization and low cost along with real-time on-site monitoring free of manual intervention [13–15]. Electronic noses facilitate the real-time measurement of ammonia concentrations in livestock and poultry breeding areas, enabling comprehensive and multi-point detection.

Generally, an electronic nose system contains four parts: a gas-collection system, sensor arrays, and signal-collection, conditioning, and data-algorithm processing modules. In this study, the gas-collection unit includes a chamber, an air pump, and a power supply. The chamber carries the sensors and has a strong influence on the gas flow into the electronic nose, thereby affecting the overall performance.

Bionic chambers that replicated the nasal-cavity structure of animals have been used in several recent electronic nose designs. For example, Chang Z. *et al*. [19] designed a bionic chamber based on the structure of the human nasal concha, the V-shaped grooves on shark skin, and the flow field distribution around the skin surface. The results indicated that the bionic chamber’s recognition rate is 5.69% higher than that of the conventional chamber when the SVM algorithm is employed to identify gas. Weng X. *et al*. [20] designed a spatial layout for sensor arrays inside an electronic nose chamber based on the distribution of olfactory receptors in a mouse nasal cavity. This bionic chamber, combined with the naïve Bayes algorithm, improved the recognition rate of the electronic nose system. Inspired by the nasal cavity structure, Liu T. *et al*. [21] designed a small-volume chamber with controllable fluidic characteristics. Experimental validation confirmed that the bionic chamber improved detection sensitivity to volatile organic compounds. In light of these earlier successes, the present study an electronic nose utilized a bionic chamber structure to detect the concentration volume of ammonia gas.

Modern data algorithms rely primarily on machine learning. The sparrow search algorithm (SSA) is a swarm-intelligence optimization algorithm proposed by Xue *et al*., inspired by the foraging and anti-predation behaviors of sparrows [22]. Compared to other common algorithms, the SSA excels in precision, convergence speed, stability, and robustness. Fan B. *et al*. [23] used near-infrared hyperspectral imaging to evaluate the effect of mutton flavor essence on mutton adulteration, employed the SSA algorithm to optimize the parameters of three models. The results indicated that the model’s efficacy is enhanced by optimizing the SSA. The SSA-SVM has an accuracy of 99.79%. Xin J. *et al*. [24] combined alternating-current magnetization with an SSA-optimized backpropagation neural network (SSA-BPNN) to identify pipeline deformations. The results showed that the SSA-BP algorithm efficiently characterized deformations within an average relative error of 10%. Li M. *et al*. [25] combined a gas sensor array with an SSA-BPNN to identify mixed harmful gases in a room, achieving an accuracy of up to 93.45%. However, none of these studies used an SSA-BPNN in combination with an electronic nose system containing a biomimetic chamber, which is adopted in this study. Accordingly, we employed an electronic nose system based on a biomimetic chamber combined with the SSA-BPNN model to classify and quantitatively predict ammonia gas concentration.

Specifically, a pump-type biomimetic electronic nose detection system is constructed in which the sensor positions within each sensor array are determined by simulating and analyzing the chamber airflow. To tackle cross-sensor interference, ethanol was used as the reference interference gas under real-world conditions. The electronic nose system was used to detect ammonia and ethanol gases. The SSA optimized the parameters of the BPNN model to classify and quantitatively predict the response data captured by the electronic nose. The predictive results based on the SSA-BPNN model are compared with predictions based on the support vector machine (SVM) and random forest (RF) models.

## 2. Materials and methods

### 2.1 Structural design of artificial olfactory system

**Fig 1:**
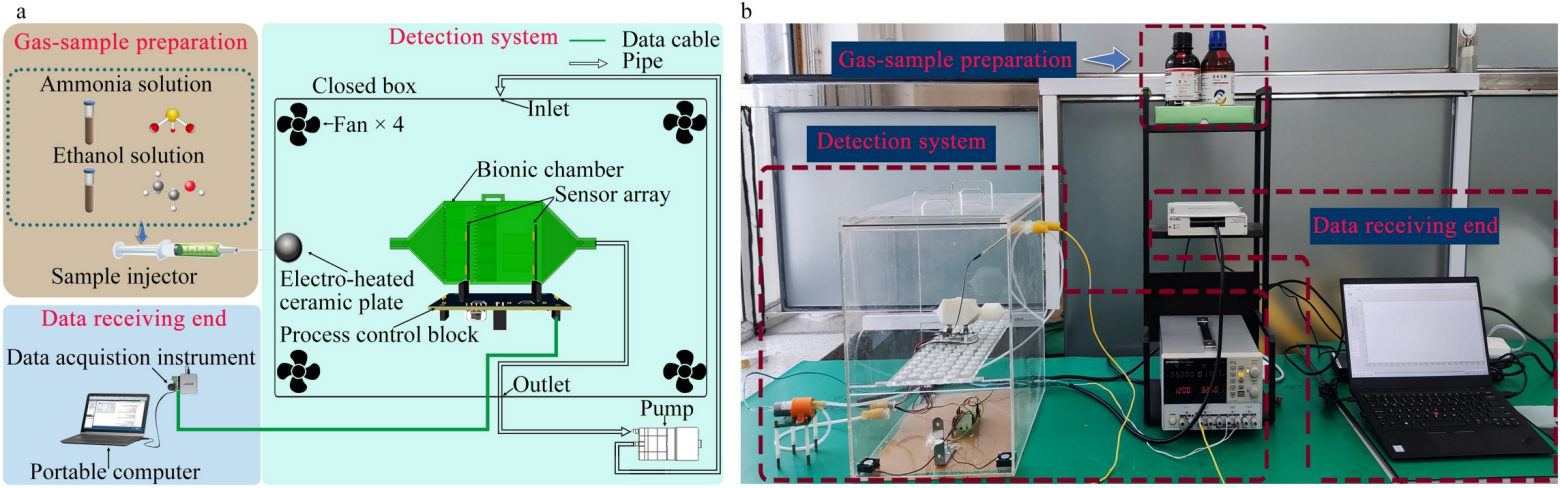
Experimental setup of the electronic nose gas detecting system: (a) schematic of the experimental framework; (b) photograph displaying the physical setup.

Fig 1 illustrates the structure of the artificial olfactory system. Fig 1(A) depicts a schematic of the experimental framework of the electronic nose system, including the gas-sample preparation module, detecting system, and data-receiving end. Fig 1(B) shows the physical setup in the laboratory. The gas-sample preparation module utilizes the rapid-evaporation property of ammonia and ethanol solutions. The detecting system comprises a closed box, fans, an electro-heated ceramic plate, sensor arrays with a bionic chamber, a main control circuit board, constant-pressure source, and gas pump. The data-receiving end comprises a portable computer and a data acquisition instrument.

The detecting system employs an active pump-type design. Through the action of the gas pump, electro-heated ceramic plate, and four fans, the substance under test swiftly evaporates and vaporizes into a gas. The volume concentration of the substance under test within the closed box remains constant. Circulating airflow is generated between the gas in the electronic-nose chamber and the closed box. The gas concentration in the circulating airflow matches that within the closed box.

### 2.2 Experimental materials and equipment

The data acquisition instrument is a National Instruments USB-6289 with a sampling frequency of 10 Hz. It is connected differentially to the output end of the sensor system. The power-supply model is a GW INSTEK GPD-4303S unit. The gas pump is a micro-diaphragm pump (Kamoer Ltd. Shanghai), model EDLP600-D12B. The pumping flow is regulated by adjusting the input voltage. The pumping flow is set at 650 SCCM. The semiconductor ammonia and ethanol sensors are MEMS sensors (Suzhou Huiwen Nano S&T Co., Ltd). These sensors encounter mutual interference when exposed to each other’s gas. The ammonia sensors (model: SMD1002) can a detect gas volume concentration in the range of 0 to 300 ppm, with a resolution of 1 ppm. The ethanol sensors (model: SMD1005) can detect a gas volume concentration in the range of 0 to 500 ppm, with a resolution of 0.2 ppm.

**Fig 2:**
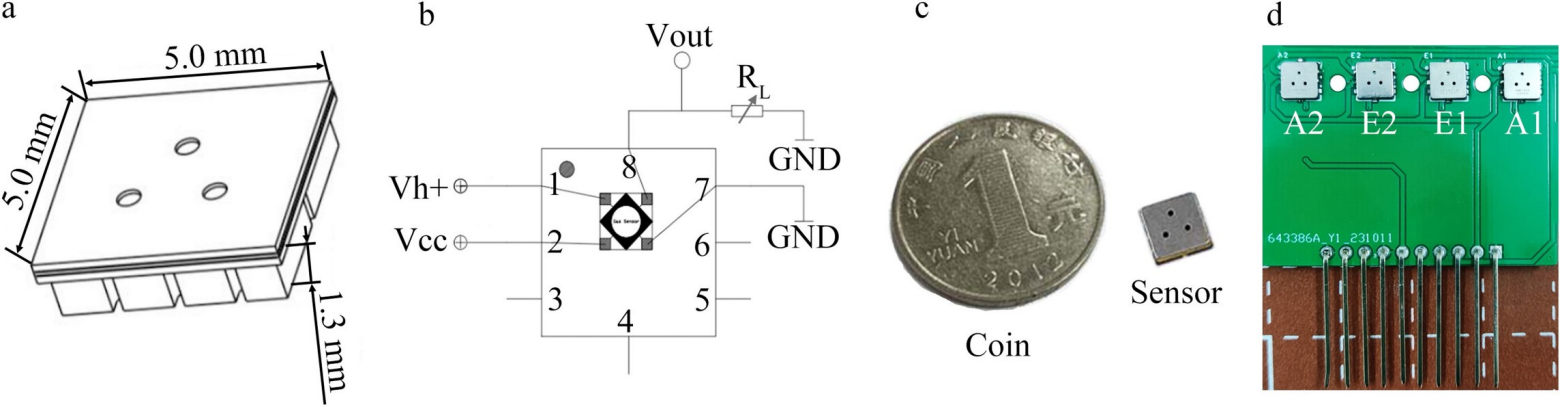
Sensor and circuit board of the electronic nose (a) Sensor packaging model (b) Internal structure and basic circuit of the sensor (c) Size comparison between the sensor and coin (d) Sensor circuit board.

Fig 2 depicts the sensor and circuit board utilized in the artificial olfactory system. Fig 2(A) displays the encapsulation model of both the ammonia and ethanol sensors. Fig 2(B) shows the internal structure and basic circuitry of these sensors, including components such as connecting wires, a silicon base, sensitive components, and supporting materials. The circuit is designed as a heating loop comprising Ports 1 and 7. Port 1 is the sensor heating port with a power supply voltage of 1.8 V, and Port 7 is grounded. Ports 2 and 8 form a measurement loop. Port 2 is a Vcc port with a power supply voltage of 5 V, and Port 8 is connected to an adjustable resistor R_L_. The ammonia sensor is designed with a maximum RL resistance of 1 MΩ, while that of the ethanol sensor can reach up to 200 kΩ. In this system, the data acquisition card is connected to the sensor ports Vout and GND, through a differential connection method. Fig 2(C) shows the physical diagram of the sensor. Fig 2(D) shows the sensor circuit board, which includes ammonia sensors (A1, A2) and ethanol sensors (E1, E2).

### 2.3 Gas preparation

The concentration of the gas volume under test was determined based on the volumes of the closed box and of the ammonia/ethanol liquids. The volume of the closed box was 48 L (0.3 × 0.4 × 0.4 m). The ammonia solution was analytically pure with an NH_3_ content of 25%. The anhydrous ethanol solution was also analytically pure with a mass fraction ≥ 99.7. Given the known volumes of the ammonia solution and liquid ethanol, the volumes of ammonia gas and ethanol gas are found from [26]

$$V_{gas}=V_{liquid}\cdot \rho \cdot 22.4/M,$$ (1)

where *V*_*gas*_ denotes the gas volume; *V*_*liquid*_ denotes the liquid volume; *ρ* denotes the relative density of the liquid; and *M* denotes the molecular weight. The volume concentration of the gas under test is the ratio of *V*_*gas*_ to the volume of the closed box.

### 2.4 Design of bionic chamber

**Fig 3:**
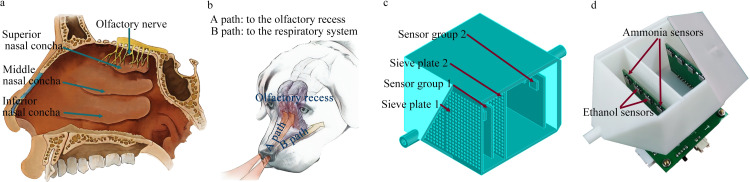
Bionic chamber design. (a) Schematic of the right sidewall of the human nasal cavity. (b) Division of the canine nasal airflow into respiratory and olfactory flows. (c) Bionic chamber model. (d) Physical appearance of the bionic chamber.

Fig 3 illustrates the design of the bionic chamber. Specifically, Fig 3(A) presents a schematic of the right sidewall of the human nasal cavity, including the distribution and structure of nasal conchae and olfactory nerves. The lateral wall of the cavity has three nasal conchae—superior, middle, and inferior [27]. The peripherals of the olfactory nerves within the mucosa of the upper part of the cavity extend through the top of the nasal cavity. The olfactory nerves originate on the surface of the posterior end of the olfactory bulb, pass through the sieve plate in the cribriform bone, and terminate in the olfactory region of the nasal cavity [28]. Fig 3(B) depicts the respiratory and olfactory nasal-airflow paths of a dog. When a dog sniffs, its olfactory airflows are directed toward the olfactory recess [29], located at the back of the nasal cavity, and the olfactory flow is diverted from the main respiratory airflow by the horizontal plate [30]. A canine’s highly developed olfactory recess generates a unique nasal airflow pattern, enhancing olfactory function and sensitivity [31]. Fig 3(C) illustrates the bionic chamber model, including the main chamber, two sets of sieve plates, and two sets of sensor arrays.

The basic design principles are as follows: (1) The sensing detection area inside the chamber is divided into two parts, i.e., sensor arrays are placed at the front and rear ends of the chamber. The sensor system can detect the volume concentration of the test gas in different areas within the chamber. (2) A sieve plate is positioned in front of each set of sensor arrays to ensure a uniform airflow in the sensor detection area [32]. (3) The sensors are placed perpendicular to the airflow at the inlet of the chamber, parallel to the sieve plate. This configuration ensures a higher lateral airflow velocity across the sensor surface. Consequently, the sensitive material can react more thoroughly with gas molecules, making the sensors more sensitive [33].

Fig 3(D) displays the physical layout of the sensor detection unit, including the bionic chamber, chamber cover plate, sensor circuit boards, and main control circuit board. The internal dimensions of the main body of the chamber are 50 × 50 × 40 mm. The outer planes of the front and rear cones are angled at 45° to the main body’s facade. The inlet and outlet both have a 3 mm diameter. The sieve plate is 1 mm thick; with 1 mm diameter holes, and the center-to-center distance between holes is 2 mm. The two sieve plates are separated by a distance of 15 mm. The bionic chamber was 3D printed using white photosensitive resin, with an accuracy of 0.1 mm. Two identical sensor-array circuit boards, each containing four sensors, are located inside the bionic chamber. The ammonia gas sensors are positioned on the left and right sides of their circuit board, whereas the ethanol sensors are placed in the middle. Ventilation holes with a diameter of 2 mm are set between adjacent sensors on each sensor circuit board. The distance between the first sensor circuit board and the first sieve plate is 12 mm, and the distance between the second sensor circuit board and the second sieve plate is 34 mm. The sensors were preheated by applying power for 48 h before use. Each experiment had a 3 min data collection period. Following each period, dry air was introduced into the electronic nose system for 10 min before the next experiment.

### 2.5 Simulation method

**Fig 4:**
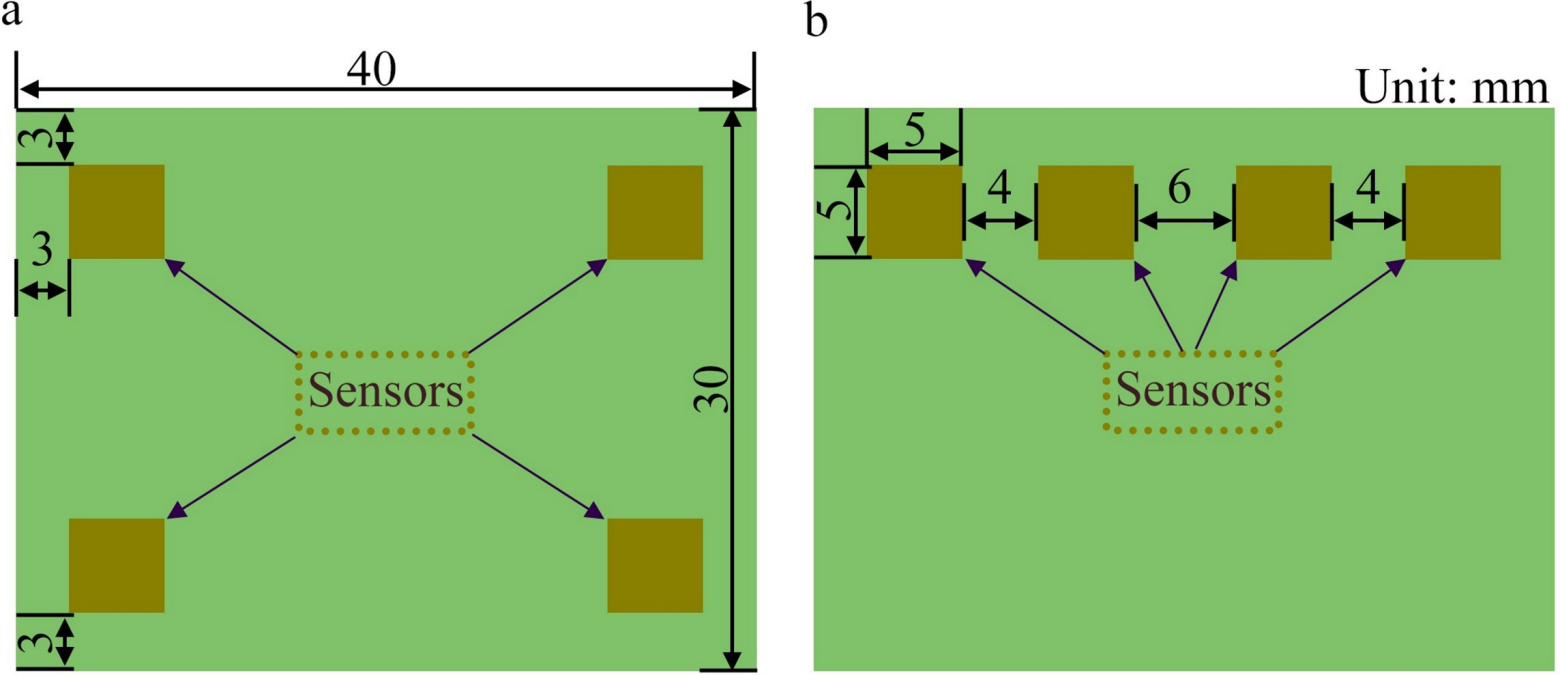
Circuit boards with two different sensor-mounting configurations, defining (a) Block A and (b) Block B.

As previously mentioned, the responses of the sensors are affected by the airflow inside the chamber. To analyze this, ANSYS software was utilized to simulate and compare the sensor response in two different mounting positions on the circuit board. Fig 4 illustrates the two sensor-placement configurations on the circuit board. In Fig 4(A), each sensor is positioned 3 mm from the edge of the circuit board, and the configuration is named “Block A.” In Fig 4(B), all four sensors are placed 3 mm from the top edge of the circuit board, the configuration is termed “Block B.”

Using AutoCAD, the structure was modeled and imported into ANSYS Workbench for fluid-simulation analysis. The simulation parameters were set as follows:

1 Velocity setting: The outlet velocity of the chamber was determined based on the formula for volume flow rate and velocity [34]:

Q=v⋅A, (2)

where *Q* denotes the volume flow rate (the pumping flow rate in the system was 650 SCCM), and *A* denotes the cross-sectional area of the pipeline (the outlet diameter of the chamber is 3 mm). The outlet velocity v was calculated to be 1.53 m/s.

2 Mathematical model: The SST k-omega mathematical model was chosen, based on the application scenario of the system.
3 Boundary conditions: The outlet velocity of the chamber was -1.53 m/s, whereas the inlet was under natural conditions.
4 Solution method: The pressure-implicit with splitting of operators (PISO) algorithm was selected as the simulation-solution method.

### 2.6 Model establishment method

The electronic nose system employed the BPNN as a data-fusion algorithm. The system utilizes eight sets of output voltage signals from ammonia and ethanol gas sensors as inputs to the BPNN. Upon training the dataset, the model generates outputs. Specifically, the classification model yields the level of volume of ammonia gas concentration in the environment, while the regression model provides the volume concentrations of both ammonia and ethanol gases.

### 2.7 SSA-based optimization of the BPNN model parameters

**Fig 5:**
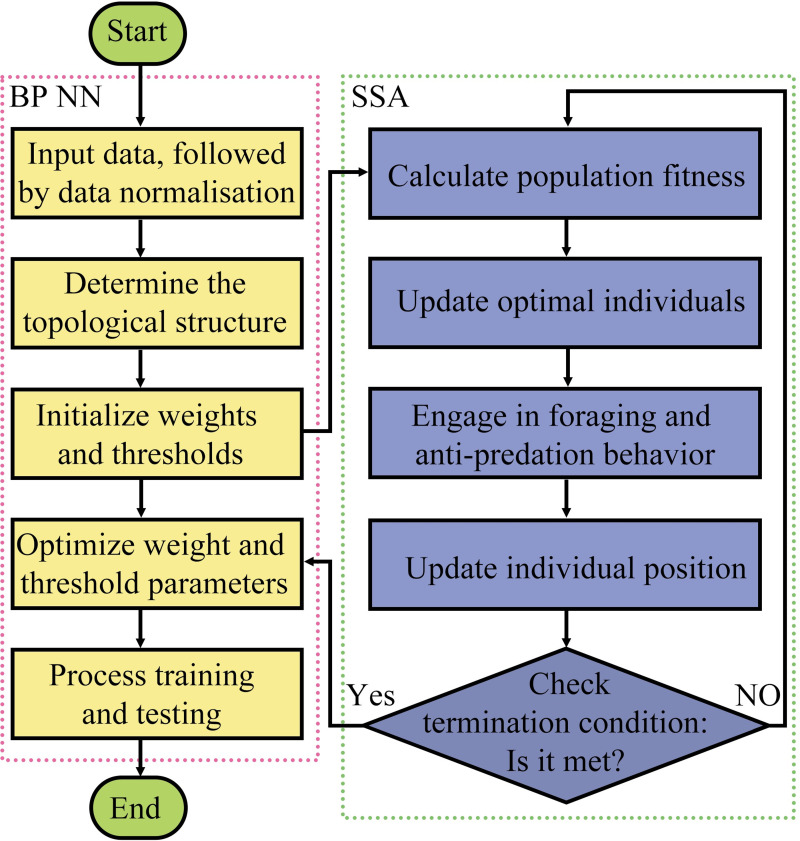
Flowchart of the SSA-based optimization of the BPNN algorithm.

The BPNN has limited global-optimization capabilities. During training, the network may converge to local optima under the effects of initial parameters, failing to find the global optima. Its poor nonlinear fitting ability results in subpar accuracy and reliability. This study used the SSA to optimize the initial weights and thresholds of the BPNN, thus enhancing the accuracy and stability of the prediction model. P. Yan et al. [35] demonstrated that the SSA, as a bio-inspired optimization algorithm, assists neural networks in escaping local optima. The SSA-BPNN algorithm’s flow is depicted in Fig 5. The main steps of the SSA-BPNN algorithm are shown in S1 File. In the optimization process, weight values are considered individuals (sparrows) within the SSA. The algorithm mimics sparrows’ foraging behavior; optimal weights, denoting the best positions of individuals, are obtained.

The initial parameter definitions for the SSA are presented in Table 1.

**Table 1:** Definitions of relevant SSA parameters.

Optimization model parameters	Defined parameter values
Maximum number of iterations *N*	50
Population size *n*	30
Number of producers *PD*	0.2×50 = 10
Number of sparrows that perceive a danger *SD*	3
Safety threshold *ST*	0.8
Alarm value *R*^2^	A random number between 0 and 1

The SSA was adopted to optimize the initial weights and thresholds of the model. The optimization algorithm program computes the mean squared error (MSE) for the training and test data to determine the SSA’s fitness value:

$$fitness=\arg\;\min(MSE_{Train}+MSE_{Test}),$$ (3)

where *MSE*_*Train*_ and *MSE*_*Test*_ represent the MSEs obtained from the training and testing processes, respectively. Lower fitness values indicate improved predictive performance of the final network on the dataset [36].

### 2.8 Model establishment and evaluation

First, classification and regression training and testing were conducted using the SSA-BPNN model. Subsequently, for comparative analysis, SVM and RF models were established and trained using the same datasets. A dataset containing 112 sets of test data was used to train the gas-analysis models. Through randomization, the dataset was divided into 78 sets of training data and 34 sets of test data. Upon establishing the models, their performances were compared based on output results and performance metrics.

## 3. Results and discussion

### 3.1 Simulation results

**Fig 6:**
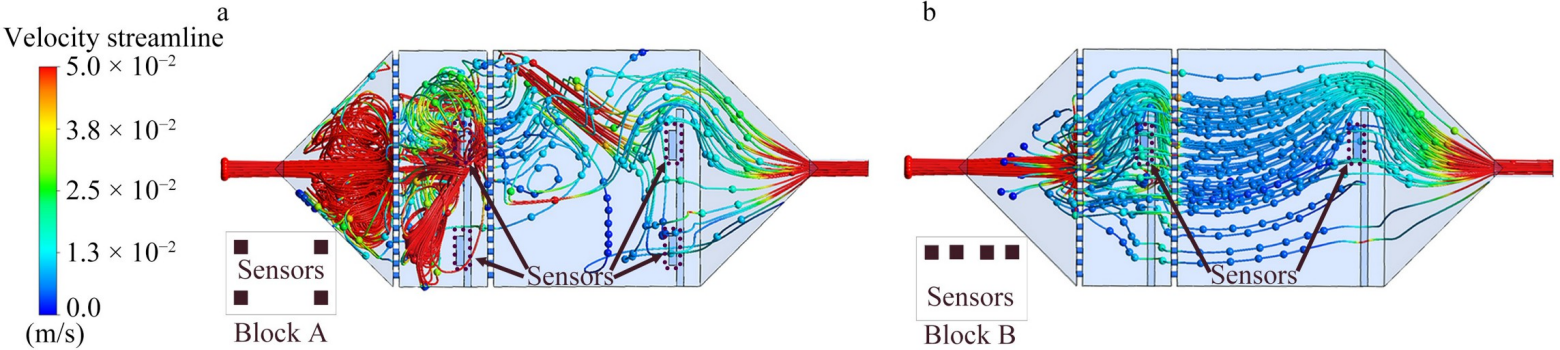
Simulation results of the gas flow velocity in bionic chambers housing (a) Block A and (b) Block B.

Fig 6 depicts the velocity-streamline diagram of airflow within identical bionic chambers containing circuit boards with two different sensor array placements. Specifically, Fig 6(A) represents Block A, while Fig 6(B) illustrates Block B. They show that the airflow reaches the highest velocity between the air inlet of the chamber and the sieve plate, forming vortices. Upon passing through the initial sieve plate, the airflow disperses, creating a more homogeneous flow field. This facilitates full contact between the sensor array and airflow. Within the chamber housing Block A, multiple vortices impeded the gas movement. Consequently, some of the gas was trapped in the chamber and thus took longer to reach the sensor surfaces. In the Block B chamber housing, fewer vortices were formed, and gases required less time to reach the sensor surfaces. Moreover, the poor gas flow near the chamber base in both scenarios rendered this region unsuitable for sensor placement. Therefore, Block B was selected as the sensor detection unit of the electronic nose.

### 3.2 Response/ recovery time of ammonia sensor

**Fig 7:**
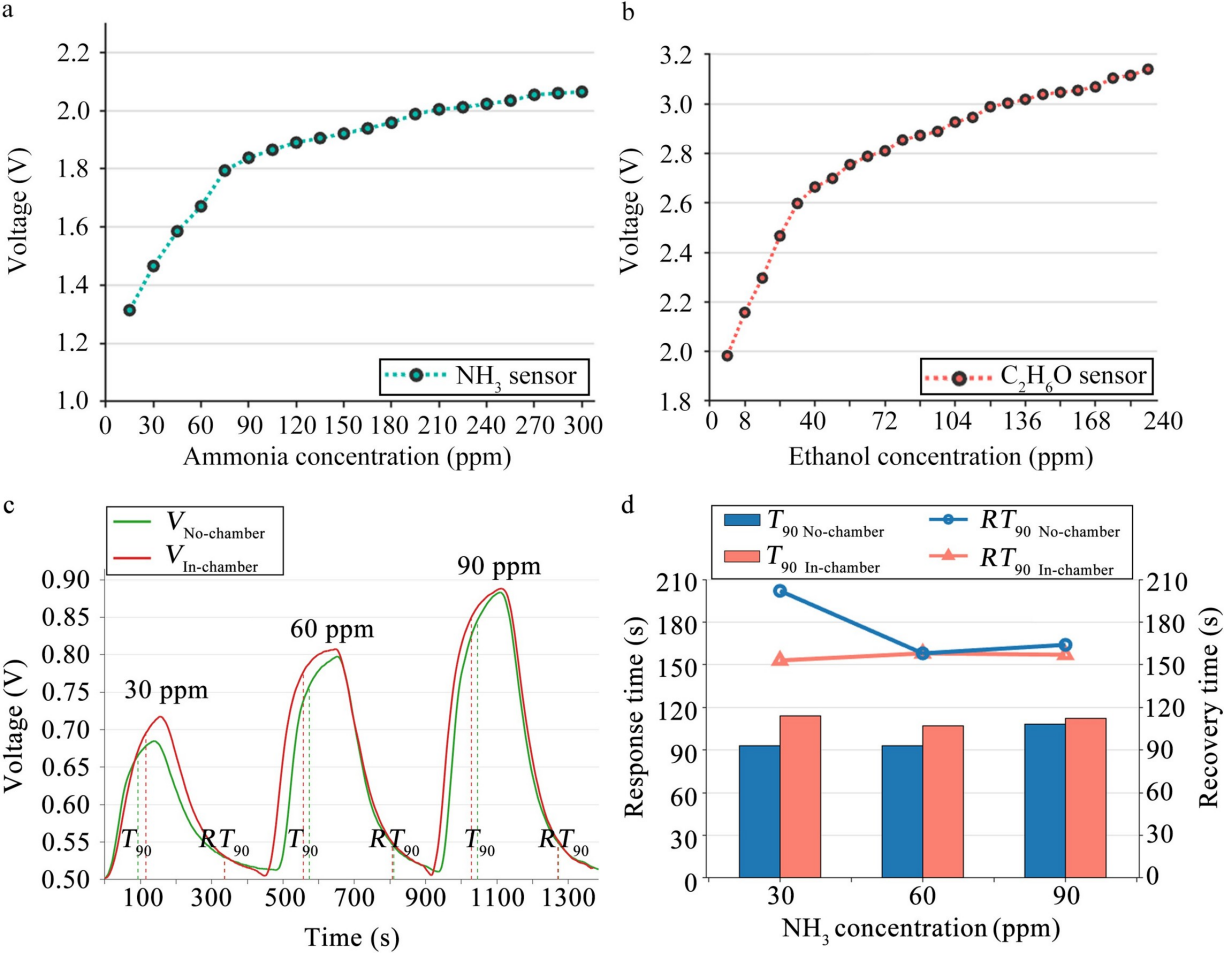
Sensor response curves and the ammonia sensor’s response and recovery time: (a) ammonia sensor’s response curve (b) ethanol sensor’s response curve (c) the relationship curve between the response/recovery voltage of the ammonia sensor and time; (ammonia concentrations are 30, 60, or 90 ppm) (d) the response/recovery time bar graph of the ammonia sensor, among them, the bar chart is the response time and the line chart is the recovery time.

The response/ recovery time describes the rate of adsorption and desorption between the sensor and the gas being measured [37]. Fig 7 shows the ammonia and ethanol sensors’ response curves and the sensors’ response and recovery times. Fig 7(A) depicts the ammonia sensor’s output voltage of 1.3–2.05 V when the ammonia concentration ranges from 15 to 300 ppm and the ethanol gas concentration is 0 ppm. Fig 7(B) depicts the ethanol gas sensor’s output voltage of 1.89–3.15 V when the ethanol gas concentration ranges from 8 to 200 ppm and ammonia gas concentration is 0 ppm. The output voltages of the ammonia and ethanol sensors increase nonlinearly throughout the entire detection interval as the gas concentration gradient increases, as shown in Fig 7(A) and 7(B). Consequently, predicting the relationship between the sensor response and the gas concentration requires a regression algorithm. Fig 7(C) and 7(D) depicts the relationship between the response voltage of the ammonia sensor and the response (*T*_90_)/ recovery (RT_90_) time when the ammonia concentrations in the closed box are 30, 60, or 90 ppm at room temperature. The figure compares the response findings of the ammonia sensor in two modes: no-chamber, where the ammonia sensor is directly exposed to the test environment, the sensor’s output voltage is represented by *V*_No-chamber_, the response time is represented by *T*_90 No-chamber_, and the recovery time is represented by RT_90 No-chamber_, and in-chamber, where the ammonia sensor is in the bionic chamber, at sensor location A2 in Fig 2(D), the output voltage of the sensor is represented by *V*_In-chamber_, the response time is represented by *T*_90 In-chamber_, and the recovery time is represented by RT_90 In-chamber_. Fig 7(C) displays that *V*_In-chamber_ > *V*_No-chamber_. This is because the airflow produced by the action of the air pump and sieve plate creates pressure on the sensor surface, rendering ammonia gas more adsorbable by the sensitive components of the sensor. The sensor then reacts more easily to ammonia, causing the system to generate a greater response voltage. Fig 7(D) presents a bar chart of the response time and a line chart of the ammonia sensor’s recovery time at three ammonia concentrations. The average response/ recovery time is: *T*_90 No-chamber_ = 98 s, *T*_90 In-chamber_ = 111 s, RT_90 No-chamber_ = 175 s, RT_90 In-chamber_ = 156 s. The response/ recovery time of the ammonia sensor indicates that: (1) When *T*_90 No-chamber_ < *T*_90 In-chamber,_ the difference in average response time is 13 s. This is because the reaction rates of sensor-sensitive materials and reactants are constant regarding time. These sensors exhibit a prolonged response time as their response voltage increases. (2) When RT_90 No-chamber_ > RT_90 In-chamber,_ the difference in average recovery time is 19 s, primarily because the air pump speeds up the entrance and outflow of air within the chamber. When compared to direct exposure to the gas being measured, the ammonia concentration near the sensor in the chamber drops dramatically and the recovery time of the sensor in the chamber is shorter. The shorter recovery time indicates that the ammonia sensor in the chamber is more efficient.

### 3.3 Sensor humidity characteristics

**Fig 8:**
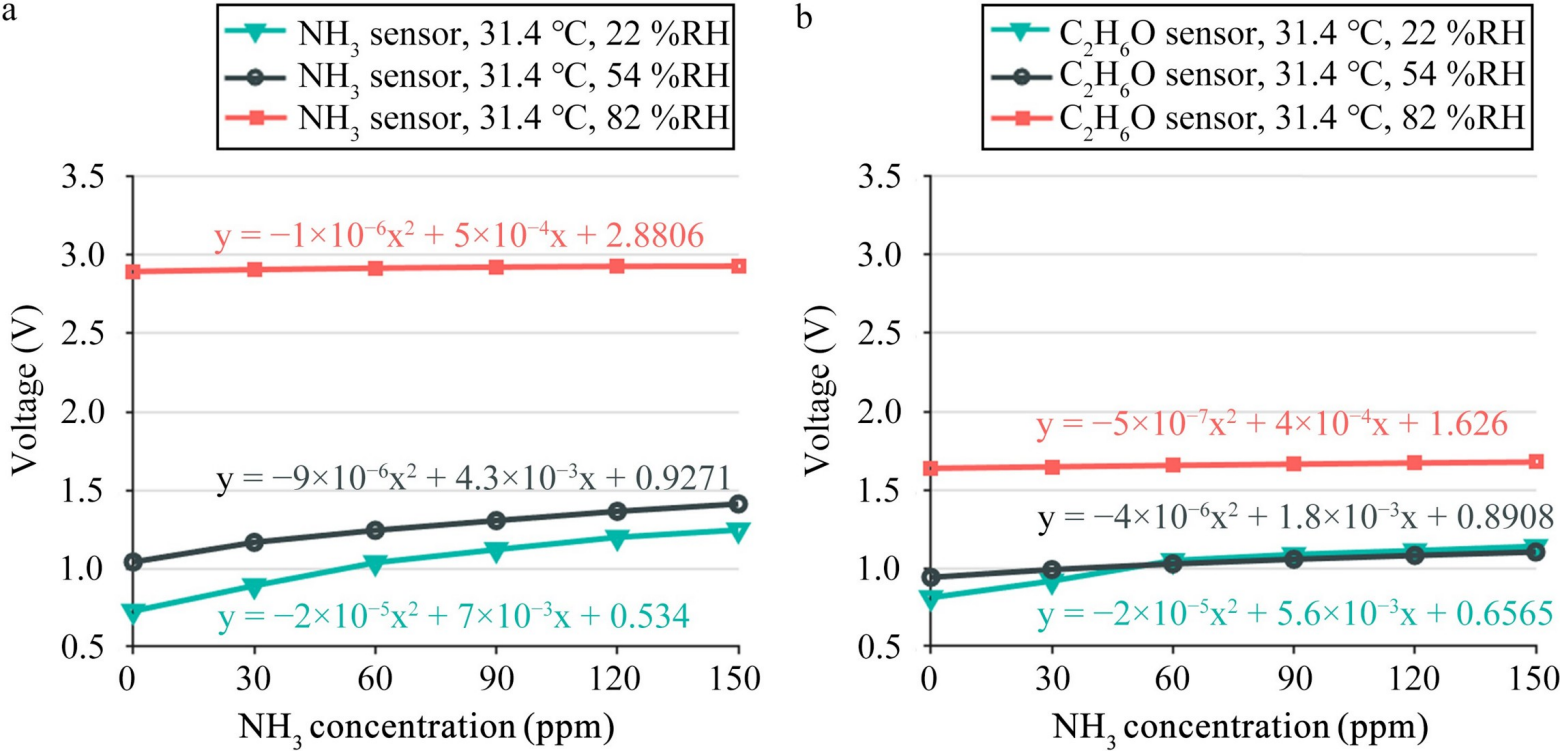
Sensor humidity characteristic curve (temperature is 31.4°C, humidity is 22, 54, and 82%RH, respectively.) (a) Ammonia sensor humidity characteristic curve. (b) Ethanol gas sensor humidity characteristic curve.

Fig 8 shows the response voltage values of the gas sensor under three different humidity conditions and six ammonia gas concentrations. Fig 8(A) depicts the response of the ammonia gas sensor, and Fig 8(B) depicts the response of the ethanol gas sensor. The temperature of the testing environment is 31.4°C, the humidity is 22, 54, and 82%RH, respectively, and the volume concentration of ammonia gas is 0, 30, 60, 90, 120 and 150 ppm, respectively. The bivariate polynomial in the figure is the trend line of the response voltage. Evidently, for the same ammonia concentration, the response voltage of the sensor increases with the humidity. Under the same humidity, the response voltage of the sensor increases with the ammonia concentration, but the rate of increase decreases. As the humidity increases, the ratio of the output voltage of ammonia and ethanol gas sensors to ammonia gas concentration decreases.

### 3.4 System response results

**Fig 9:**
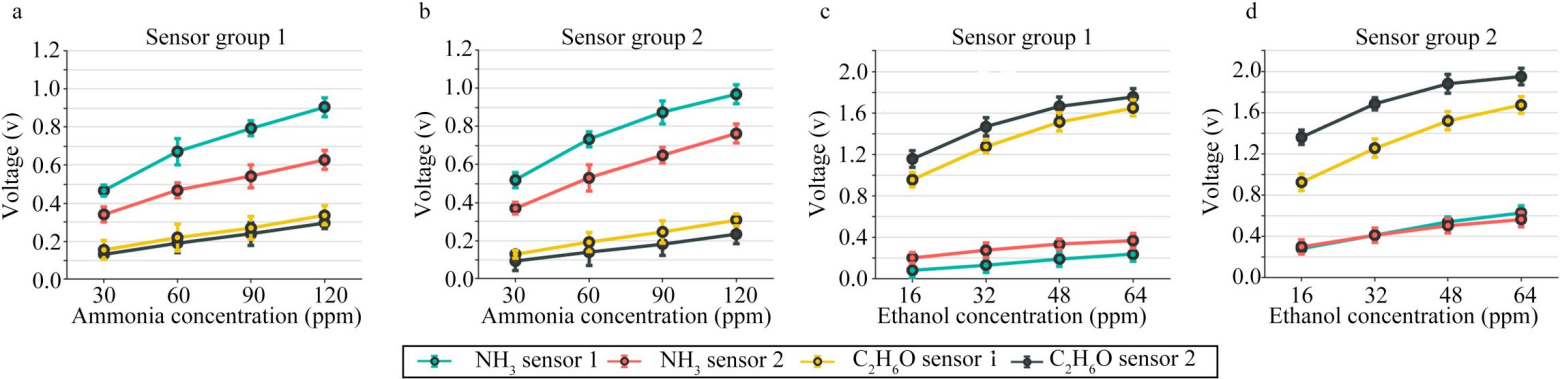
Relationship between sensor output voltage and gas concentration: (a) Output voltage of the four sensors in Sensor group 1 regarding the ammonia concentration. (b) Output voltage of the four sensors in Sensor group 2 regarding the ammonia concentration. (c) Output voltage of the four sensors in Sensor group 1 regarding the ethanol concentration. (d) Output voltage of the four sensors in Sensor group 2 regarding the ethanol concentration.

The semiconductor gas sensor is created by the oxidation-reduction reaction of gas on the semiconductor surface, which changes the resistance value of the sensitive element [38]. The response voltage of the sensor utilized in this system is calculated using the change in resistance value of the sensitive element. As sample gases passed through the bionic chamber, the electronic nose system witnessed changes in the output voltage. The moving average approach was employed in MATLAB to preprocess the response voltage of the electronic nose, with a smoothing value of 0.2. The response voltage of the sensor stationary signal is averaged over 10 s to 20 s, starting two minutes after the gas to be detected enters the closed box. Fig 9 shows the relationship between sensor output voltage, gas concentration, and the sensor response deviation. In subgraphs (a) and (b), the volume concentration of ethanol gas is 0 ppm, whereas in subgraphs (c) and (d), the volume concentration of ammonia gas is 0 ppm. The sensor has a relative mean deviation of 1.45%. The output curves in the figure show that the response voltage increases as the volume concentration of the ammonia and ethanol gases increase. Different gas sensors have varying responsiveness to ammonia and even identical sensors may yield different response signals because of variance deviations in the gas-sensitive material during fabrication and parameter errors in peripheral circuit components. Ammonia sensors exhibit good responsiveness to ammonia, but ethanol gas also causes changes in their output. Similarly, both ethanol and ammonia can induce changes in the output voltage of ethanol sensors. Owing to the poor selectivity and stability of gas sensors, it is difficult to derive precise response equations. Therefore, the cross-sensitivity of the gas sensor arrays was employed in combination with the BPNN algorithm to distinguish the ammonia concentration levels in mixed gases and predict the ammonia concentration.

### 3.5 Multiclass classification of ammonia concentration based on the SSA-BPNN

**Fig 10:**
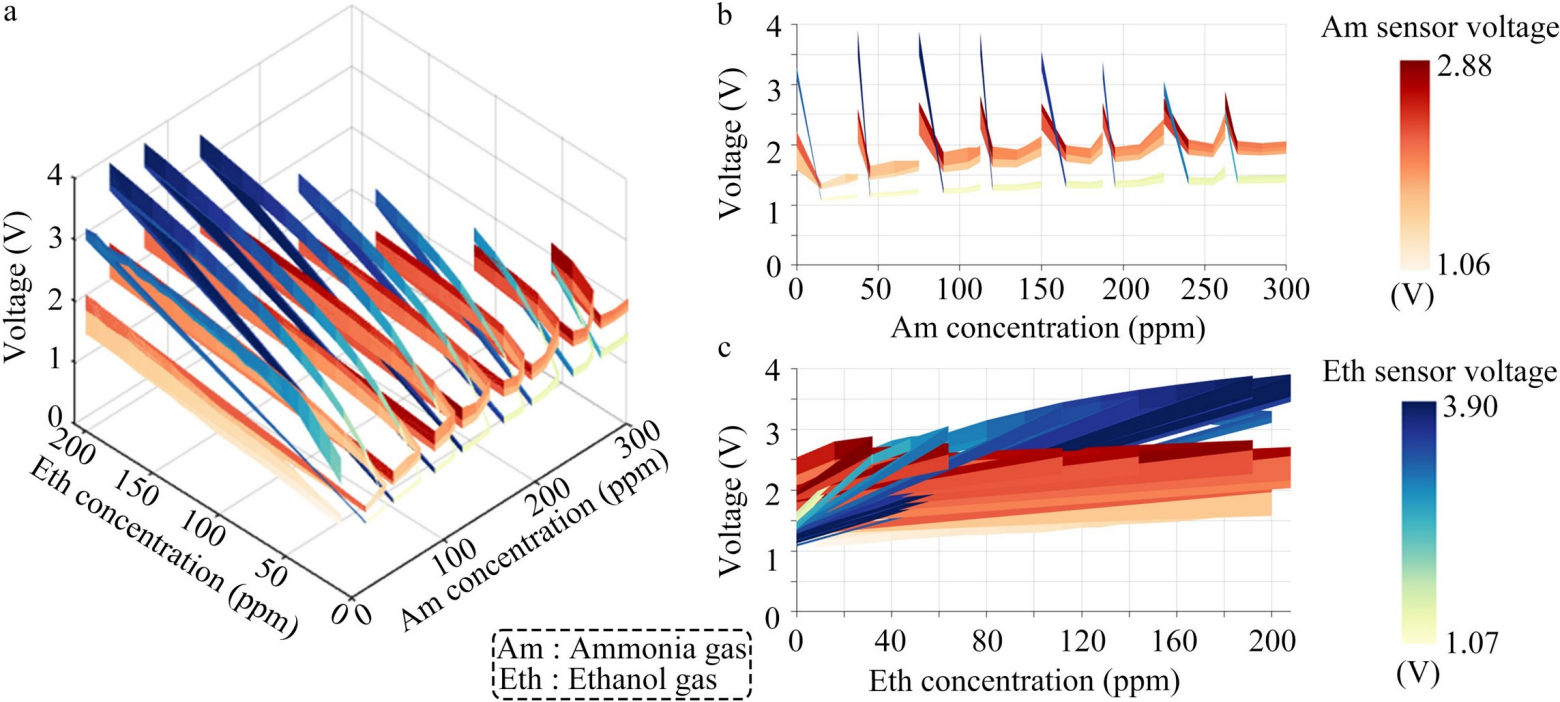
Gas sensing response results of the ammonia and ethanol gas sensors to ammonia and ethanol gases. (a) Three-dimensional (3D) view. (b) Ammonia concentration and response voltage. (c) Ethanol gas concentration and response voltage.

Fig 10 shows the gas sensing response results of the ammonia and ethanol gas sensors to ammonia and ethanol gases. For each type of sensor, the sensor response forms a surface. Fig 10(A) shows a three denominational plot with the X and Y axis representing ammonia and ethanol gas concentrations, and Z representing the output voltage. Fig 10(B) presents the ammonia concentration and sensor response voltage. The detection range of the ammonia sensor is 0–300 ppm. Fig 10(C) presents the ethanol gas concentration and response voltage. The detection range of the ethanol gas sensor is 0–240 ppm. The figure shows that the ammonia and ethanol sensors react to both ammonia and ethanol gases. The volume concentration of ammonia gas is 30–240 ppm in the overlapping area of sensor response; the volume concentration of ethanol gas is 50–100 ppm. Consequently, the sensor response data are categorized and recognized using an intelligent identification method.

**Fig 11:**
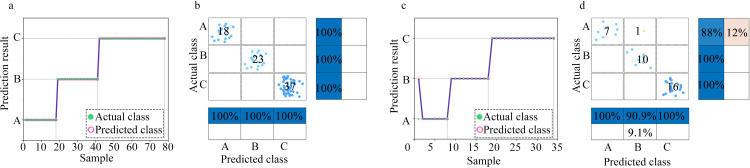
SSA-BPNN classification results: (a) Scatter plot of the predicted classes based on the training set. (b) Confusion matrix for the training set. (c) Scatter plot of the predicted classes based on the test set. (d) Confusion matrix for the test set.

**Fig 12:**
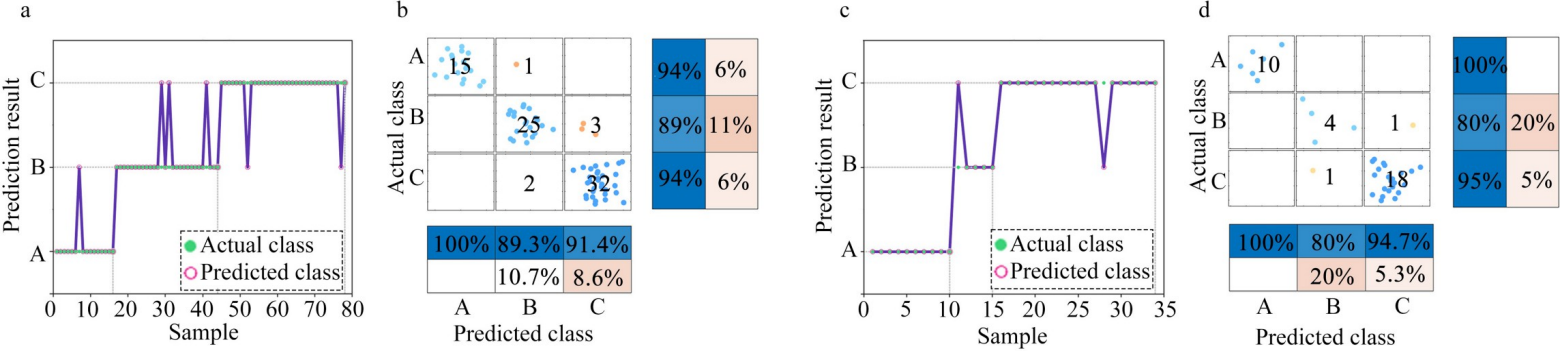
SVM classification results: (a) Scatter plot of the predicted classes based on the training set. (b) Confusion matrix for the training set. (c) Scatter plot of the predicted classes based on the test set. (d) Confusion matrix for the test set.

**Fig 13:**
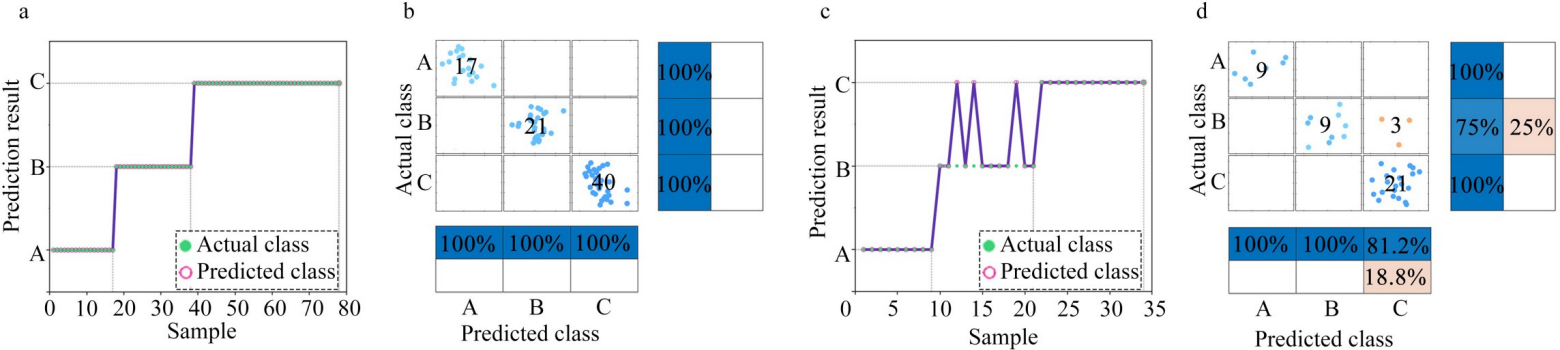
RF classification results: (a) Scatter plot of the predicted classes based on the training set. (b) Confusion matrix for the training set. (c) Scatter plot of the predicted classes based on the test set. (d) Confusion matrix for the test set.

When establishing the classification model, we considered the degree to which environmental ammonia impacts on humans and animals, as discussed in the first paragraph of the paper. Accordingly, three classes were defined: Class A–ammonia concentration < 25 ppm; Class B–ammonia concentration > 25 and < 100 ppm; and Class C–ammonia concentration > 100 ppm. Figs 11–13 respectively display the classes predicted by the SSA-BPNN, SVM, and RF models for ammonia concentration levels. The subgraph (a) presents the scatter plots for the classes predicted based on the training set. The subgraph (b) provides the confusion matrices for the training set. The subgraph (c) presents the scatter plots for the classes predicted based on the test set and the subgraph (d) provides the confusion matrices for the test set. Regarding the prediction results for the ammonia concentration levels, using the SSA-BPNN model, produced seven errors on the test set in the predicted classes. The SVM model produced six errors on the training set and two errors on the test set. The RF model produced three errors on the test set.

Table 2 presents the classification accuracies of the models. The classification accuracy of the SSA-optimized BPNN model is higher than those of the SVM and RF models. With the SSA-BPNN model, the classification accuracy for the training set was 100%, and for the test set, 97.1%. This indicates that the pump-type electronic nose system with a bionic chamber, when used with the SSA-BPNN model to predict the volume concentration levels of ammonia gas, has the required level of accuracy.

**Table 2:** Classification accuracies of the three models.

Model	Accuracy	
	Training set	Test set
SSA-BPNN	100%	97.1%
SVM	92.2%	94.1%
RF	100%	91.2%

### 3.6 Regression prediction of ammonia concentration based on the SSA-BPNN model

**Fig 14:**
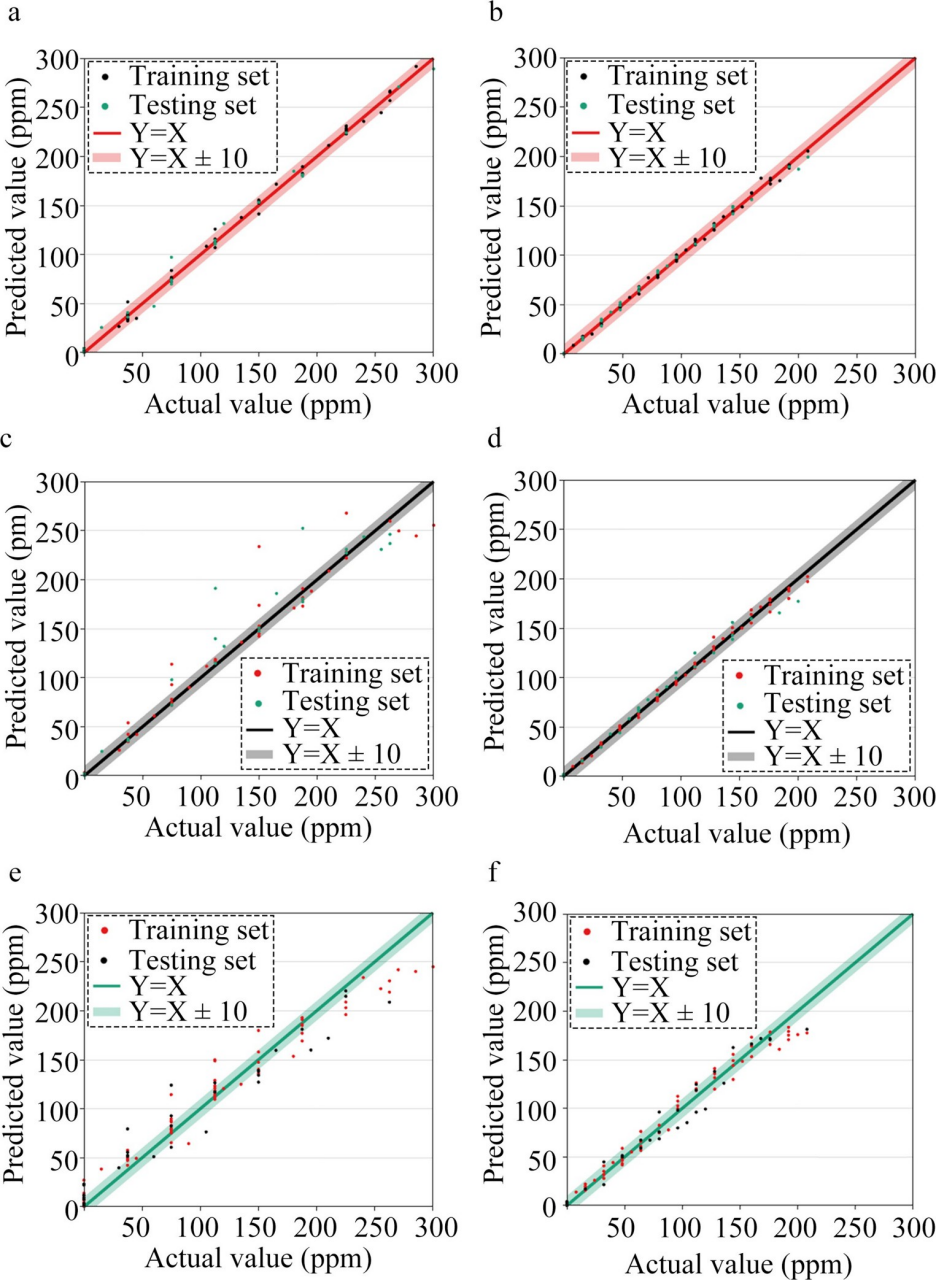
Relationship between actual and predicted gas volume concentrations for training and test datasets: (a) Ammonia gas results using the SSA-BPNN model. (b) Ethanol gas results using the SSA-BPNN model. (c) Ammonia gas results using the SVM model. (d) Ethanol gas results using the SVM model (e) Ammonia gas results using the RF model. (f) Ethanol gas results using the RF model.

The sample data was fed into the SSA-BPNN, SVM, and RF models for training and testing, obtaining the predictive results of the three models. Fig 14 illustrates the relationship between the actual and predicted gas volume concentrations, where subgraphs (a) and (b) present the ammonia and ethanol predictions using the SSA-BPNN model. Subgraphs (c) and (d) depict the ammonia and ethanol predictions using the SVM model, and subgraphs naïve and (f) present the predictions using the RF model. The ammonia gas volume concentration ranged from 0 to 300 ppm, while the ethanol gas volume concentration ranged from 0 to 208 ppm.

The predictive results of the three models are better for ethanol than for ammonia, potentially owing to the mismatch between the gas concentrations and the optimal sensor detection range. The maximum concentration of ammonia had already reached the maximum ammonia-sensor detection limit of 300 ppm, whereas the maximum concentration of ethanol at 208 ppm is far below the maximum ethanol-sensor detection limit of 500 ppm. Unlike the predicted-vs-actual-value scatter plots generated by the SVM and RF models, that generated by the SSA-BPNN model for ammonia concentration reveals that the majority of results fall within the range of Y = X±10. Thus, the BPNN model optimized via the SSA demonstrates a significantly better predictive accuracy for ammonia concentration compared to SVM and RF models. Moreover, the number of predictions deviating from the actual values by more than 10 ppm is much lower for the SSA-BPNN model than for the SVM and RF models.

**Fig 15:**
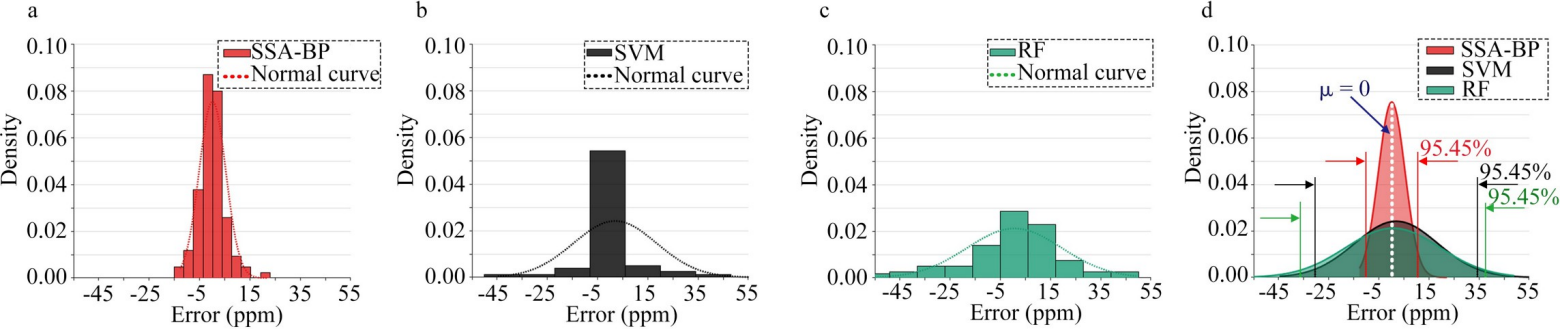
Statistical comparison of absolute errors between predicted and actual ammonia values: errors using (a) the SSA-BPNN model, (b) SVM model, and (c) RF model. (d) Probability density function of the normal distribution of absolute errors generated by the three models.

**Fig 16:**
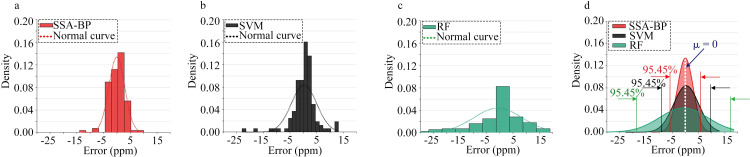
Statistical comparison of absolute errors between the predicted and actual ethanol values: errors obtained from (a) the SSA-BPNN model, (b) SVM model, and (c) RF model. (d) Probability density function of the normal distribution of absolute errors generated by the three models.

Figs 15 and 16 depict the probability density of absolute errors in predicting the volume concentrations of ammonia and ethanol gases using the SSA-BPNN, SVM, and RF models. Herein, the X-axis represents the absolute error, the Y-axis represents the probability density, and the density function is defined as

$$f(x)=\frac{1}{\sqrt{2\pi \sigma }}e^{-\frac{{\left(x-\mu \right)}^{2}}{2\sigma^{2}}},-\infty <x<\infty$$ (4)

Figs 15(D) and 16(D) show that the distribution of absolute errors is more concentrated with the SSA-BPNN model than with the SVM and RF models. The standard deviations of the normal distributions of prediction errors for ammonia concentration are 4.28, 12.62, and 15.21 for SSA-BPNN, SVM, and RF models, respectively. For ethanol concentration, the standard deviations are 2.47, 3.77, and 7.85, respectively. The SSA-BPNN model achieves a narrower range of prediction errors than the SVM and RF models, thereby reducing the overall prediction error and enhancing prediction accuracy.

Table 3 presents a comparison of the SSA-BPNN, SVM, and RF models in terms of three evaluation metrics—coefficient of determination (R^2^), mean absolute error (MAE), and root mean square error (RMSE). The R^2^ value of the SSA-BPNN model surpasses 0.99, exceeding those of the SVM and RF models; this indicates a higher degree of fit for the SSA-BPNN regression model. Additionally, the MAE and RMSE values of the SSA-BPNN model are smaller than those of the SVM and RF models, which indicates that for the SSA-BPNN, minimal differences exist between the predicted and actual values and little susceptibility to residual outliers, signifying superior performance. Thus, among the three models, the optimal one for predicting gas volume concentrations is the SSA-BPNN model, which boasts the best fit and least error.

**Table 3:** Comparison of electronic nose based on bionic chamber.

Bionic design of chamber	Sensor type	Classification		Regression		Reference
		Algorithm	Accuracy rate	Algorithm	R^2^	
Human nasal cavity structure, canine olfactory recess	MEMS	SSA-BPNN	99.1%	SSA-BPNN	> 0.99	This work
Human turbinate structure, V-groove structure on shark skin surface, and flow field distribution around the skin surface.	Semiconductor	SVM	93.14%	—	—	[19]
Distribution of olfactory cells in mice	Semiconductor	Naive Bayes	92.3%	—	—	[20]
Canine ethmoturbinal, canine olfactory recess	Electrochemistry	—	—	BPNN	> 0.98	[32]

SSA enhanced the predictive accuracy and stability of the BPNN for quantitative analysis, surpassing the SVM and RF models in predictive performance. The system is highly accurate within a certain error range, enabling the precise quantitative detection of ammonia gas.

The results demonstrate the excellent fitting capability of the SSA-BPNN algorithm. It can avoid selective sensitivity to target gases caused by the cross-sensitivity of gas sensors; thus, it enhances the performance of the system in both qualitative and quantitative analysis. Coupled with a bionic chamber based electronic nose system, the algorithm demonstrates the ability to improve the precision and accuracy of target-gas detection.

### 3.7 Comparison of electronic nose systems

The SSA-BPNN model, in conjunction with the bionic chamber-based electronic nose presented in this work, enables the detection of mixed gases containing ethanol and ammonia with good sensitivity. The electronic nose system employed in this investigation and the detection outcomes in other studies are compared in Tables 3 and 4.

**Table 4:** Comparison of detection results of the SSA optimization algorithm.

Detection method	Classification		Regression		Reference
	Algorithm	Accuracy rate	Algorithm	R^2^	
Electronic nose	SSA-BPNN	99.1%	SSA-BPNN	0.99	This work
Hyperspectral imaging	SSA-SVM	99.9%	SSA-SVM	0.94	[23]
Alternating current magnetization	—	—	SSA-BPNN	0.99	[24]
Electronic nose	SSA-BPNN	93.45%	SSA-BPNN	0.98	[25]

Table 3 presents a comparison of gas detection outcomes obtained from an electronic nose that utilizes a bionic chamber. The spatial layout of the electronic nose chamber and sensor was designed by references [19, 20], respectively. They used an unoptimized algorithm and achieved accuracies of 93.14% and 92.3%, respectively. In [31], a double-chamber construction with a sieve plate was constructed for the electrochemical sensor based on its volume size. The BP algorithm was employed to predict the outcome, resulting in an R^2^ value of 0.98. This work utilizes the knowledge of the human nasal cavity structure and the characteristics of olfactory airflow in dogs to create a spatial arrangement for two sets of sensor arrays with sieve plates. The BP algorithm, optimized by the SSA, is employed in this design. The detection findings have a classification accuracy of 99.1%, and the regression prediction R^2^ exceeds 0.99.

Table 4 presents a comparison of detection outcomes obtained from a machine learning algorithm that has been optimized using the SSA method. In [23], the SSA-SVM approach is employed for both classification and regression prediction. The classification accuracy achieved is 99.9%, while the R^2^ value for regression prediction is 0.94. Both Reference [24, 25] utilized the SSA-BPNN for prediction. The R^2^ values for regression prediction are 0.99 and 0.98, respectively. The findings of this study, along with the three studies, demonstrate that the model outcomes are enhanced by optimizing the model parameters using SSA.

In this study, under the joint action of the electronic nose bionic chamber and bionic optimization algorithm SSA-BPNN, the classification prediction accuracy of electronic nose system is high and the regression prediction R^2^ exceeds 0.99. To achieve this success rate, the role of the bionic chamber is more important owing to the following:

- The algorithm is based on training data collected using sensors with chambers.
- The electronic nose system adopts the active pumping intake mode, which makes the gas flow in the space, the gas concentration near the sensor is stable, and the output voltage of the sensor exhibits less fluctuation.
- The sieve plate in the chamber enables the airflow to form a vortex in the chamber, which enhances contact with the gas for optimal measurements.

The algorithm is also critical. Apart from the SVM and RF models, we used conventional models (such as Radial Basis Function), the BP algorithm without optimization, and other BP optimization algorithms (such as GA-BP) to train using data. However, the success rate of the other models did not match that of the SSA-BPNN model.

## 4. Conclusions

This study employed an active pump-type electronic nose system equipped with a bionic chamber to classify and quantitatively analyze ammonia gas volume concentrations. Sensor placement was based on the velocity streamlines within the chamber. The response recovery time of an ammonia sensor was evaluated at three different gas concentrations. By optimizing the BPNN model parameters using the SSA, a model was established for predicting ammonia gas volume concentrations.

The results show that the sensor has a relative mean deviation of 1.45%. The ammonia sensor’s output voltage is 1.3–2.05 V when the ammonia concentration ranges from 15 to 300 ppm. The ethanol gas sensor’s output voltage is 1.89–3.15 V when the ethanol gas concentration ranges from 8 to 200 ppm. The electronic nose ammonia sensor’s response time is 111 s, and the recovery time is 156 s. The average response time of the ammonia sensor in the chamber is 13 s slower than that of the sensor directly exposed to the gas being measured, while the average recovery time is 19 s faster. The SSA-BPNN model achieves an accuracy of 99.1%, surpassing the accuracy of the SVM and RF models. The SSA-BPNN model achieved an R^2^ value (> 0.99), which was higher than that of the SVM and RF models. Moreover, it outperformed the other models in terms of the absolute error, MAE, and RMSE. Thus, the electronic nose combined with the bionic chamber and the SSA-BPNN model possesses excellent detection capabilities for both the ammonia and ethanol gas mixtures. The proposed system enables accurate, rapid, and stable detection of ammonia concentrations in livestock- and poultry-breeding facilities. Notably, this study examined only one interfering gas (ethanol), and other interfering gases e.g. hydrogen sulfide, should be investigated in the future. Measurements of more gas concentration values will be made in the future, and the SSA-BPNN algorithm will be applied to learn and recognize the response of the electronic nose. The aim is to improve the pattern recognition system’s capacity for generalization. Relative humidity affects the sensor response when measuring gas sensing at room temperature. Subsequently, the sensor signals will be measured in different relative humidity environments and an intelligent algorithm will compensate for the gas concentration values.

## Supporting information

S1 File The main steps of the SSA-BPNN algorithm. (PDF)
