# Supplementary material for: Ammonia and ethanol detection via an electronic nose utilizing a bionic chamber and a sparrow search algorithm–optimized backpropagation neural network
Source: PLoS One. 2024 Dec 3;19(12):e0309228. doi: 10.1371/journal.pone.0309228 (PMC11614243; doi:10.1371/journal.pone.0309228)
Supplement: S1 File — (PDF) [file pone.0309228.s001.pdf]

The main steps of the SSA-BPNN algorithm are as follows:

Step 1: Initialize position and fitness function for the sparrow population, setting initial values for algorithm parameters, such as the number of iterations (N), population size (n), number of producers (PD), number of sparrows who perceive the danger (SD), safety threshold (ST), and alarm value (R^2).

Step 2: Commence iterative cycles.

Step 3: Sort the population to obtain the best sparrow positions and their corresponding fitness.

Step 4: Update producer positions using

$$X_{i,j}^{t+1} = \begin{cases} X_{i,j}^{t} \bullet \exp\left(\dfrac{-i}{a \bullet N}\right) & \text{if } R_2 < ST \\ X_{i,j}^{t} + Q \bullet L & \text{if } R_2 \geq ST \end{cases}, \tag{3}$$

where t denotes the current number of iterations; X_{ij} is an element of a high-dimensional array that provides the position of the i-th sparrow along the j-th dimension; Q is a random number conforming to a normal distribution; L is a $1 \times d$ unit row vector; a is a random number within the range [0, 1].

Step 5: Update scrounger positions using

$$X_{i,j}^{t+1} = \begin{cases} Q \bullet \exp\left(\dfrac{X_{worst} - X_{i,j}^{t}}{i^2}\right) & \text{if } i > n/2 \\ X_P^{t+1} + \left| X_{i,j}^{t} - X_P^{t+1} \right| \bullet A^{+} \bullet L & otherwise \end{cases}, \tag{4}$$

where X_p denotes the current foraging location of the producer within the population; X_{worst} denotes the poorest foraging position within the population, i.e., the position of the sparrow with the lowest fitness; A is a $1 \times D$ matrix containing elements randomly assigned as 1 and –1, yielding $A^{+}=A^{T}(AA^{T})^{-1}$.

Step 6: Include anti-predation behavior by updating the positions of the sparrows with

$$X_{i,j}^{t+1} = \begin{cases} X_{best}^{t} + \beta \bullet \left| X_{i,j}^{t} - X_{best}^{t} \right| & \text{if } f_i > f_{best} \\ X_{i,j}^{t} + K \bullet \left(\dfrac{\left| X_{i,j}^{t} - X_{worst}^{t} \right|}{(f_i - f_{worst}) + \varepsilon} \right) & \text{if } f_i = f_{best} \end{cases}, \quad (5)$$

where β is a random number that follows a normal distribution, serving to regulate the intervals for updating positions; K is a random number within the range [–1, 1]; f_i is the fitness value for each sparrow at the current iteration; ε signifies a minimum constant aimed at preventing a zero denominator.

Step 7: Update the historically best fitness.

Step 8: Iteratively execute Steps 3–7 until the current number of iterations reaches N, concluding the loop; output the highest fitness and its corresponding individual position.
